# Nicotinic cholinergic regulation of olfactory bulb blood flow response in aged rats

**DOI:** 10.1186/s12576-022-00859-9

**Published:** 2023-03-02

**Authors:** Sae Uchida, Jura Moriya, Daichi Morihara, Fusako Kagitani

**Affiliations:** 1 Department of Autonomic Neuroscience, Tokyo Metropolitan Institute of Gerontology, 35-2 Sakaecho, Itabashi-Ku, Tokyo, 173-0015 Japan; 2 Division of Applied Biological Chemistry, Graduate School of Agriculture, Tokyo University of Agriculture and Technology, Fuchu, Japan; 3 Department of Applied Biological Science, Faculty of Agriculture, Tokyo University of Agriculture and Technology, Fuchu, Japan

**Keywords:** Aging, Olfactory bulb, Cerebral blood flow, Olfactory nerve stimulation, Nicotinic acetylcholine receptors, Rats

## Abstract

In our previous research, we had demonstrated the crucial role of neuronal nicotinic acetylcholine receptors (nAChRs) in potentiation of the olfactory bulb blood flow response to olfactory stimulation in adult rats. The present study examined the effects of nAChR activation on the olfactory bulb blood flow response in rats aged 24–27 months. We found that, under urethane anesthesia, unilateral olfactory nerve stimulation (300 μA, 20 Hz, 5 s) increased blood flow within the ipsilateral olfactory bulb, without changes in the systemic arterial pressure. The increase in blood flow was dependent upon the current and frequency of the stimulus. Intravenous administration of nicotine (30 μg/kg) had little effect on the olfactory bulb blood flow response to nerve stimulation at either 2 Hz or 20 Hz. These results suggest a reduction in nAChR-mediated potentiation of the olfactory bulb blood flow response in aged rats.

## Introduction

A decline in olfactory function is a common aspect of normal aging [1–4]. It is also an early symptom of Alzheimer’s disease [5–7]. The olfactory bulb, the first processing station of olfactory information in the brain, receives cholinergic basal forebrain input, as does the neocortex contributing cognitive function [8, 9].

Rodent studies by our laboratory have shown that nicotinic acetylcholine receptors (nAChRs) in the brain play a crucial role in vasodilation, increasing the regional blood flow in the neocortex, induced by basal forebrain cholinergic activation or nicotine injection [10–14]. Activation of the α4β2 subtype of nAChRs was found to be responsible for nicotine-induced neocortical vasodilation [15]. When compared to adult rats of 3-10 months, this vasodilation was relatively well-maintained in old rats of 23-26 months, but showed a marked decline in very old rats of 32-36 months [14].

Our studies also elucidated the role of nicotinic cholinergic transmission in the olfactory bulb in adult rats [16, 17]. Activation of α4β2 nAChRs by nicotine injection triggers the potentiation of olfactory bulb vasodilation induced by olfactory stimulation [16]. This indicates that activation of α4β2 nAChRs in the brain plays a role in increasing the sensitivity of the olfactory bulb to olfactory stimuli. It is important to know aging effects on nicotinic cholinergic regulation of the olfactory bulb blood flow response. Thus, the present study aimed to investigate the effects of nicotine stimulation of nAChRs on the olfactory bulb vasodilation induced by olfactory stimulation in old rats of 24–27 months. We will discuss these aging effects by comparing the present results in old rats with our previous reports in adult rats [17].

## Methods

### Experimental animals

The experiments in this study were performed on five male Fischer rats (360–460 g; 24–27 months old). Our research was conducted in accordance with the Guidelines for Proper Conduct of Animal Experiments (established by the Science Council of Japan in 2006) and was approved by the Animal Care and Use Committee of the Tokyo Metropolitan Institute of Gerontology.

### General surgery and anesthesia

The rats were anesthetized subcutaneously with urethane (1.4 g/kg), after initial inhalation of 4.2% sevoflurane for approximately 5 min. Respiration was maintained using an artificial respirator (model 683, Harvard, USA) via a tracheal cannula. The end-tidal CO_2_ concentration was monitored using a gas monitor (Microcap, Oridion Medical, Jerusalem, Israel), and was maintained at 3.0%–4.0% by controlling respiratory volume and frequency. Arterial blood pressure was measured through a catheter inserted into a femoral artery with a pressure transducer (TP-400 T, Nihon Kohden, Tokyo, Japan). Body temperature was measured rectally and continuously using a thermistor and maintained at approximately 37.5 °C using a body temperature control system (ATB-1100, Nihon Kohden). Anesthesia was sustained with additional urethane doses (100 mg/kg, i.v. via a catheter inserted into a femoral vein) when necessary. This was determined by monitoring body movement, blood pressure stability, and respiratory movement.

### Measurement of regional blood flow in the olfactory bulb and neocortex

Each animal was mounted on a stereotactic instrument (SR-5R-HT, Narishige, Co., Ltd., Tokyo, Japan) in a prone position. Regional cerebral blood flow was measured using laser speckle contrast imaging, as has been previously described [17–19].

Briefly, a craniotomy was performed and the surface of the brain was then covered with mineral oil, followed by a glass coverslip. For the laser speckle contrast imaging, we used a Moor full-field perfusion imaging device consisting of an infrared laser diode (785 nm wavelength) and a CCD camera (FLPI-2, Moor Instruments, Devon, UK). The imaging device was fixed, and the zoom was adjusted to cover the dorsal surface of the brain from the most anterior part of the olfactory bulb to the frontal cortex. The viewing field covered approximately 108 mm^2^ (12 mm × 9 mm) with a matrix of 150 × 116 pixels, providing an approximate resolution of 79 μm per pixel. The images were sampled at 25 Hz.

To analyze spatial changes in blood flow, the acquired images were further averaged over 1-s time bins. The baseline image, which was obtained just before olfactory nerve stimulation (− 1 to 0 s), was then subtracted from the other images to assess the relative blood flow changes. To quantify temporal blood flow changes (in arbitrary units), time courses were extracted for three regions of interest, which were selected on the imaging device screen using 1.0-mm-diameter circles. These were positioned bilaterally, avoiding visible blood vessels, in the area of the olfactory bulb (anterior–posterior [AP] = 7.0–8.0 mm from bregma, lateral [L] = 0.6–1.6 mm to the midline), and the frontal cortex (AP = 1.0–4.0 mm, L = 1.0–4.0 mm) [20, 21] ipsilateral to the side of olfactory nerve stimulation.

### Stimulation of the olfactory nerve

The unilateral olfactory nerve was electrically stimulated as has been previously described [17, 22]. Briefly, a coaxial metal electrode with an outer diameter of 0.2 mm was stereotaxically inserted into the olfactory nerve bundle approximately 4 mm posterior to the nasofrontal suture. Electrical stimulation of the olfactory nerve was performed using a stimulator (SEN-3301, Nihon Kohden) and a stimulus isolation unit (SS-202 J, Nihon Kohden). Repetitive electrical square pulse stimuli of 0.5 ms in width, with varying intensities (20–400 μA) and frequencies (0.5–200 Hz), were applied for 5 s. To examine the relationships between the intensities and frequencies of the stimulation and the magnitudes of the olfactory bulb blood flow response, the stimuli were applied with increment increases, going from low to high intensity at steady frequency (20 Hz), and from low to high frequency at steady intensity (300 μA or 400 μA). The minimum interval between stimuli was 1.5 min. To examine the effect of nicotine on the olfactory nerve stimulation-induced blood flow response, the parameters of electrical stimulation were set at an intensity of 300 μA or 400 μA with two different frequencies (2 and 20 Hz). The order of these two frequencies was random, and the minimum interval between stimuli was 2 min.

### Drug administration

(−) Nicotine (Tokyo Kasei Kogyo, Tokyo, Japan) was diluted in saline to a final concentration of 30 μg/kg body weight (calculated as the freebase). This was then injected slowly (over approximately 1 min) into the femoral vein on the rat. We chose a nicotine dose of 30 μg/kg because our previous report found this to be optimal for stimulating nAChRs in the brain parenchyma while keeping changes in systemic arterial pressure to a minimum [14]. We also previously found it to effectively enhance the olfactory bulb blood flow responses to olfactory stimulation in adult rats [16, 17].

### Hypercapnic stimulation

Hypercapnia was induced in the rats by introducing 5% CO_2_ with air balanced gas via the inspiratory gas for 30 s, as previously described [23, 24].

### Data collection and statistical analysis

The analog signals obtained that measured regional blood flow and systemic arterial pressure were recorded on a desktop computer using an analog-to-digital (A/D) converter (Micro 1401 mkII, Cambridge Electronic Design, Cambridge, UK) and Spike 2 software (Spike 2, Cambridge, UK) that enabled offline analyses.

All values were presented as mean ± SEM. Changes in the regional blood flow and the mean arterial pressure evoked by olfactory nerve or hypercapnic stimulation were assessed by a Friedman’s test followed by Dunn’s multiple comparison test. Changes in regional blood flow responses to olfactory nerve stimulation before and after nicotine injection were compared using Wilcoxon matched-pairs signed rank test. A *p*-value of < 0.05 was considered statistically significant.

## Results

### Spatial and temporal changes in cerebral blood flow resulting from olfactory nerve stimulation in aged rats

**Fig. 1:**
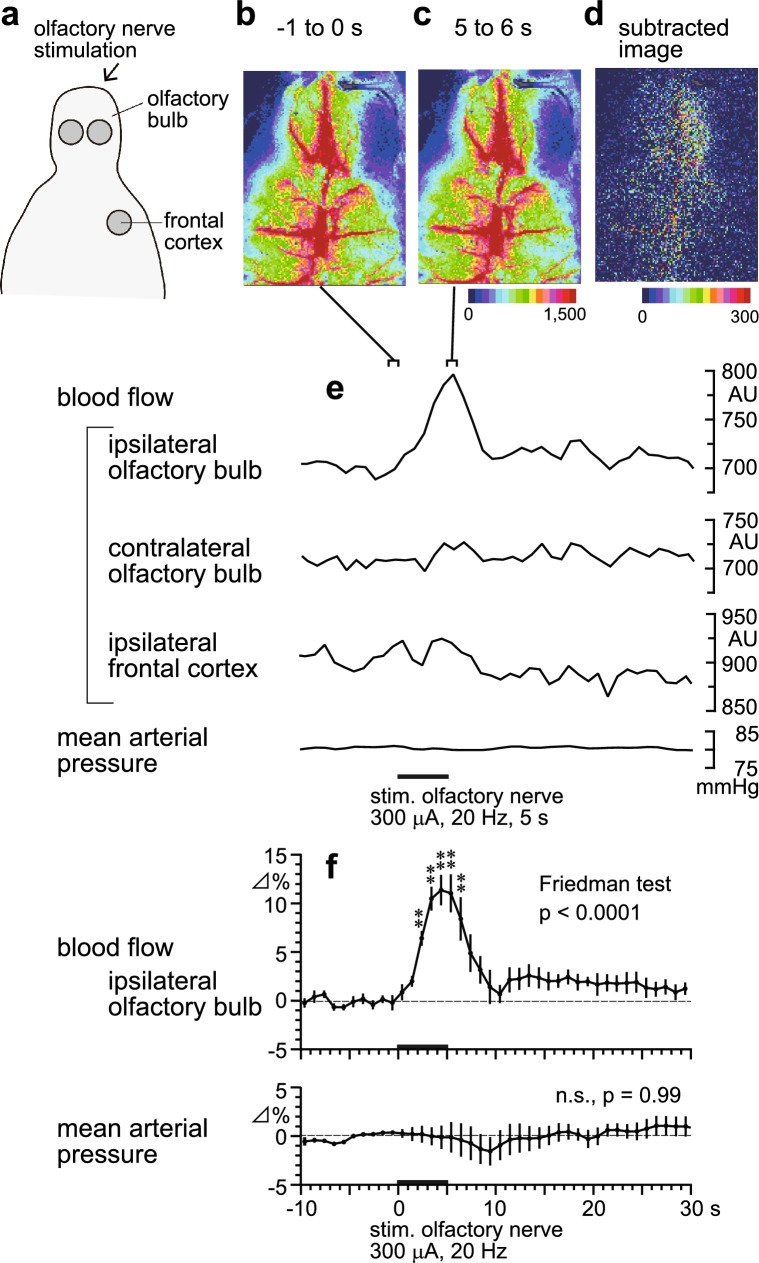
Spatiotemporal changes in regional cerebral blood flow following olfactory nerve stimulation in aged rats. **a** Schema showing the area of blood flow recorded using a laser speckle contrast imaging device. **b**–**e** Sample recordings of regional cerebral blood flow and mean arterial pressure in one aged rat. **b, c** Averaged signal over selected 1-s period. **d** Differential signal changes were obtained from b and c by subtracting the baseline signal from the subsequent image (b: − 1 to 0 s, c: 5–6 s). **e** Sample recordings of the mean arterial pressure and the regional cerebral blood flow responses to olfactory nerve stimulation ipsilateral and contralateral olfactory bulb, and the ipsilateral frontal cortex (the region of interest from which data were extracted are indicated by the *grey circles* in **a**). **f** Signal changes (as percentages) in the ipsilateral olfactory bulb blood flow and the mean arterial pressure in response to olfactory nerve stimulation, averaged every 1 s. Changes in each parameter were expressed as the percentages of the corresponding basal values (the mean of the values from the 10 s before time zero). Each point and vertical bar represents a mean ± SEM (*n* = 5). *p < 0.05, **p < 0.01: significantly different from the mean pre-stimulus basal value (− 1 to 0 s), determined with a Friedman’s test followed by Dunn’s multiple comparison test

Changes in the cerebral blood flow and mean arterial pressure induced by olfactory nerve stimulation at 300 μA and 20 Hz for 5 s were measured in aged rats (Fig. 1). Unilateral stimulation of the olfactory nerve increased olfactory bulb blood flow on the side ipsilateral to the stimulation (Friedman test, *p* < 0.0001). This blood flow usually began to increase approximately 1 s after stimulation onset (Fig. 1e). The increased blood flow became statistically significant 2–3 s after stimulation onset and reached a maximum of 11% ± 2% (*n* = 5) near the end of the stimulation period (Fig. 1f). In contrast, changes in blood flow within the contralateral olfactory bulb, and ipsilateral frontal cortex to olfactory nerve stimulation, were negligible (Fig. 1e) and statistically insignificant (Friedman test, *p* = 0.11 for contralateral olfactory bulb, *p* = 0.59 for ipsilateral frontal cortex). Each rat’s resting arterial pressure was measured before the stimulation was applied. The mean resting pressure was 78 ± 4 mmHg (*n* = 5). Blood pressure was not influenced by olfactory nerve stimulation (Figs. 1e, f, Friedman test, *p* = 0.99).

### Changes in blood flow in response to olfactory nerve stimulation in aged rats

**Fig. 2:**
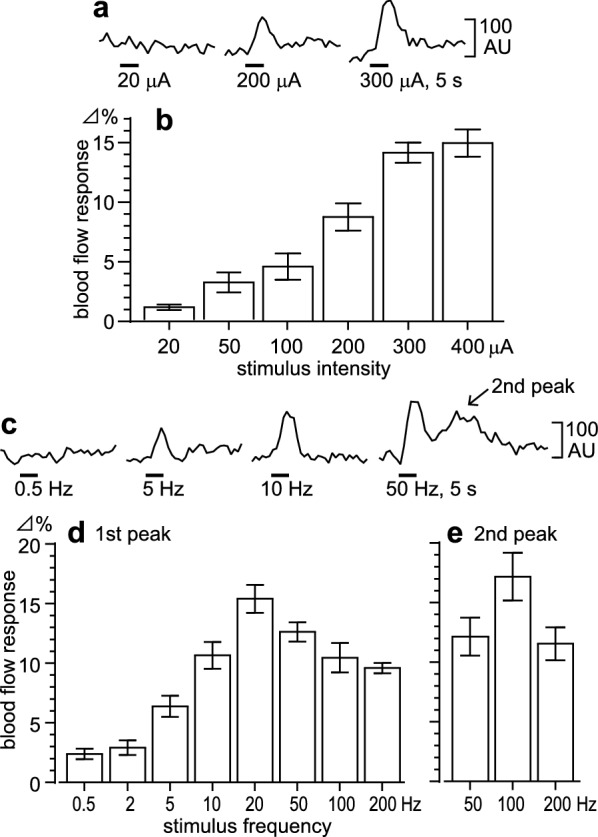
Olfactory bulb blood flow responses to olfactory nerve stimulation in aged rats. The effects of various intensities (**a**–**b**) and frequencies (**c**–**e**) of electrical stimulation of the unilateral olfactory nerve on regional olfactory bulb blood flow on the side ipsilateral to the stimulus. **a**–**b** Stimulus frequency, 20 Hz. **c** Stimulus intensity, 400 μA. **d**–**e** Stimulus intensity, 300 or 400 μA. **a, c** Sample recordings of regional blood flow in the olfactory bulb. **b, d, e** Maximum levels of regional olfactory bulb blood flow. These occurred within 8 s (first peak) or 15–22 s (second peak) of stimulation onset. These are expressed as the percentage of the pre-stimulus basal blood flow (the mean of the values recorded in the 10 s before time zero), by which blood flow was increased. Each column and vertical bar represents a mean ± SEM (*n* = 5)

The effects of electrical stimulation of the unilateral olfactory nerve on regional blood flow in the olfactory bulb were examined, using different stimulus intensities (Fig. 2a, b) and frequencies (Fig. 2c–e), and focusing on the blood flow in the side ipsilateral to the stimulation. When the frequency of the stimulation was kept at a constant of 20 Hz, olfactory bulb blood flow was increased in response to intensities of ≥ 100 μA in a current-dependent manner in all 5 rats tested. The blood flow responses were greatest with intensities of 300 and 400 μA (Fig. 2a, b). When the intensity of the stimulus was kept at a constant of either 300 μA or 400 μA, olfactory bulb blood flow was increased in response to frequencies of  ≥ 5–200 Hz in all 5 rats tested (Fig. 2c, d). The blood flow response was greatest with a frequency of 20 Hz. Stimulation with higher frequencies of 50–200 Hz produced a second peak blood flow response, approximately 15–22 s after the onset of olfactory nerve stimulation (Fig. 2c, e).

### Effect of intravenous nicotine on the olfactory bulb blood flow response to olfactory nerve stimulation in aged rats

The effect of nicotine on the olfactory bulb blood flow response to olfactory nerve stimulation with frequencies of 2 Hz (subthreshold frequency) and 20 Hz (the maximum frequency for the first peak) and a constant intensity of 300 μA or 400 μA was examined. Before nicotine injection, the olfactory bulb blood flow responses elicited by olfactory nerve stimulation using these two different frequencies were confirmed to be stable between the two trials.

**Fig. 3:**
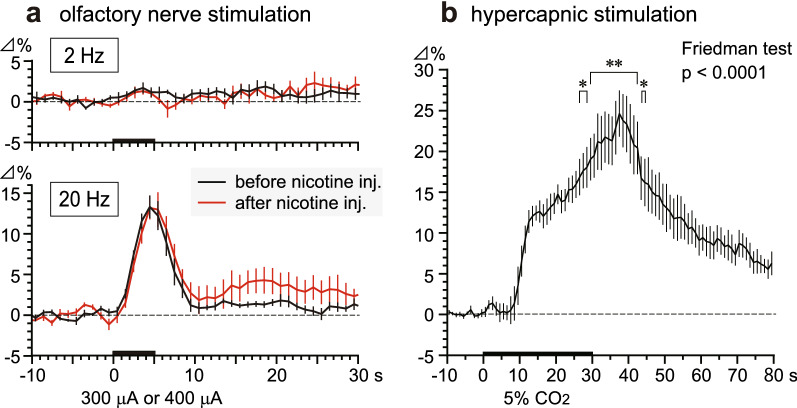
Changes in the olfactory bulb blood flow following nicotine administration or hypercapnia in aged rats. **a** Graph of the time course of regional blood flow responses in the olfactory bulb ipsilateral to the olfactory nerve stimulation. The blood flow values were averaged every 1 s before (black line) and after (red line) nicotine injection (30 μg/kg). The stimulus frequencies used were 2 Hz (upper graphs) and 20 Hz (lower graphs). Changes in regional blood flow are expressed as percentages of the corresponding basal values (the mean value from those recorded over the 10 s before time zero). Each point and vertical bar represent a mean ± SEM (*n* = 5). **b** Graph of olfactory bulb regional blood flow response time courses, averaged every 1 s (*n* = 5). *p < 0.05, **p < 0.01: significantly different from the mean pre-stimulus basal value (− 1 to 0 s), determined with a Friedman’s test followed by Dunn’s multiple comparison test

Figure 3a shows the time course of the olfactory bulb blood flow responses to olfactory nerve stimulation using these two frequencies, before (black line) and at 3–11 min after nicotine administration (30 μg/kg, red line) in five rats. Nicotine produced little effect on the olfactory bulb blood flow responses to olfactory nerve stimulation with frequencies of 2 Hz and 20 Hz. Stimulation with a frequency of 2 Hz produced no obvious changes in blood flow either before or after nicotine injection (Fig. 3a, top). Stimulation with a frequency of 20 Hz produced same increases in olfactory bulb blood flow, with the response being marginally potentiated by nicotine injection in three of the five rats tested. With 20 Hz stimulation, the peak increment was similar in both conditions (before or after nicotine injection), but the subsequent blood flow was marginally greater after nicotine injection than before (Fig. 3a, bottom). However, the olfactory bulb blood flow response to 20 Hz stimulation between these two conditions were not statistically significant (*p* = 0.31, tested by Wilcoxon matched-pairs signed rank test), when the averaged blood flow values for a 30-s period after stimulation onset were compared (before vs after nicotine injection, 3.01 ± 0.38% vs 4.39 ± 1.11%). Neither the basal olfactory bulb blood flow nor the mean arterial pressure was significantly changed by nicotine administration.

### Effect of hypercapnic stimulation on the olfactory bulb blood flow in aged rats

Figure 3b depicts the time course of the olfactory bulb blood flow responses to hypercapnic stimulation of the olfactory bulbs of five old rats. We found olfactory bulb blood flow to be significantly increased by hypercapnia (inhalation of 5% CO_2_ for 30 s) reaching a maximum of 25% ± 3% (Friedman test, *p* < 0.0001).

## Discussion

**Fig. 4:**
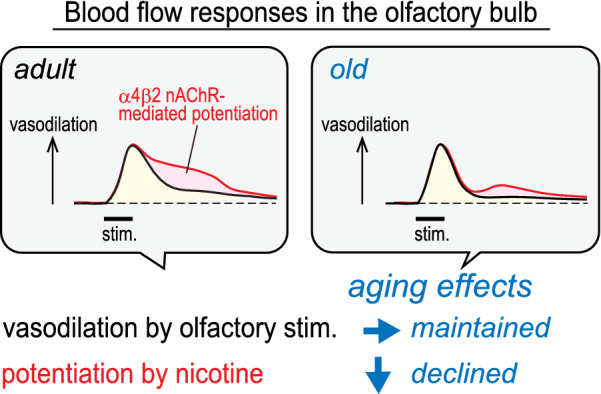
Schematic diagram showing aging effects on α4β2 nAChR-mediated potentiation of vasodilation within the olfactory bulb. nAChR, nicotinic acetylcholine receptor

This study has demonstrated that olfactory nerve stimulation produces vasodilation in the olfactory bulb. However, the nicotine-induced potentiation of olfactory bulb vasodilation due to α4β2 nAChR activation was found to have diminished considerably in old rats of 24–27 months (Fig. 4).

### Comparison of the effects of aging on the blood flow responses of the neocortex and the olfactory bulb

In our previous study, we showed that α4β2 nAChR-mediated vasodilation in the neocortex induced by nicotine injection is relatively well-maintained in old rats of 23–26 months but markedly declines in very old rats of 32–36 months [14, 15]. In the present study, we showed that the α4β2 nAChR-mediated potentiation of olfactory bulb vasodilation induced by nicotine injection is reduced in old rats of 24–27 months. Based on these results, we posit that age-related impairment of α4β2 nAChR function may affect the olfactory bulb earlier than the neocortex. The nAChR-mediated regulation of olfactory function may be a useful marker for the early detection of impairments in olfaction and cognition.

### Olfactory bulb vasodilation sensitivity to olfactory nerve stimulation in aged rats

We found that electrical stimulation of the unilateral olfactory nerve in old rats produced an increase in olfactory bulb blood flow ipsilateral to the side of stimulation. Blood pressure was unaffected. The blood flow increase in the ipsilateral olfactory bulb depended on the current and frequency of the stimulus. The spatiotemporal blood flow response characteristics and the current and frequency dependence of prompt vasodilation of the olfactory bulb were the same as those previously observed in adult rats [17]. This is consistent with research by Kass et al. [25] who demonstrated that odor-evoked synaptic output from the olfactory sensory neurons to the olfactory bulb glomeruli is relatively stable in anesthetized mice throughout normal aging, from 6 to 24 months old.

### The decline of nicotine effects on the olfactory bulb in aged rats

Our previous study with adult rats showed intravenous injection of nicotine to increase olfactory bulb sensitivity to olfactory stimulation and the potentiation of regional vasodilation [16, 17]. This potentiation effects was found to be due to the activation of α4β2 nAChRs in the brain, since an α4β2-preferring nAChR antagonist (dihydro-β-erythroidine) abolished the potentiation effects [16]. In the present study, the potentiating effects of nicotine on olfactory bulb vasodilatory responses to olfactory nerve stimulation were greatly reduced in old rats of 24–27 months. No nicotine-induced potentiation was observed in the blood flow response to 2 Hz and 20 Hz nerve stimulation. In contrast, olfactory bulb vasodilatory response to hypercapnic stimulation, indicating the vasodilatory ability of the olfactory bulb, was considerably greater than its response to olfactory nerve stimulation (Fig. 3). We propose that, with age, the olfactory bulb blood vessels maintain their vasodilatory ability but reactivity to nicotine diminishes. Since α4β2 nAChRs are responsible for the effects of nicotine, described in our research, a decline in α4β2 nAChR function in old rats of 24–27 months may be the cause of this (Fig. 4).

In rat brains, α4 and β2 mRNA levels are known to decrease from 7–29 months of age, with further decreases at 32 months. This is relatively constant across in different areas of the brain [26]. Decreases in α4 and β2 mRNA levels as well as α4β2 nAChR availability have also been reported in the human brain with normal aging [27] and Alzheimer’s disease [28]. The diminished olfactory bulb blood flow potentiation effects of nicotine through α4β2 nAChR activation in old rats may be due to this decline in α4β2 nAChRs in the brain [26–28]. Thus, age-related decline in α4β2 nAChR function may be the mechanism responsible for the reduction in olfactory sensitivity in old rats of 25 months [29], and may also cause olfactory dysfunction in older peoples and those with Alzheimer’s disease [1, 6, 30, 31].

## Conclusion

This study demonstrated that α4β2 nAChR-mediated potentiation of the olfactory bulb vasodilation response to olfactory stimulation declines in old rats of 24–27 months. These findings broaden the present understanding of the importance of α4β2 nAChRs in the maintenance of olfactory function.

## Data Availability

The data that support the findings of this study are available from the corresponding author upon reasonable request.

## Abbreviation

nAChR: Nicotinic acetylcholine receptor
